# Psychostimulant-induced aberrant DNA methylation in an in vitro model of human peripheral blood mononuclear cells

**DOI:** 10.1186/s13148-022-01303-w

**Published:** 2022-07-16

**Authors:** Kaili Anier, Kelli Somelar, Külli Jaako, Margret Alttoa, Kerli Sikk, Raul Kokassaar, Kai Kisand, Anti Kalda

**Affiliations:** 1 Department of Pharmacology, Institute of Biomedicine and Translational Medicine, University of Tartu, Ravila 19, 50411 Tartu, Estonia; 2 Department of Molecular Pathology, Institute of Biomedicine and Translational Medicine, University of Tartu, Ravila 19, 50411 Tartu, Estonia

**Keywords:** DNA methylation, DNA demethylation, Amphetamine, Cocaine, Decitabine, Human peripheral mononuclear cells

## Abstract

**Background:**

Several reports have provided crucial evidence in animal models that epigenetic modifications, such as DNA methylation, may be involved in psychostimulant-induced stable changes at the cellular level in the brain. Epigenetic editors DNA methyltransferases (DNMTs) and ten-eleven translocation enzymes (TETs) coordinate expression of gene networks, which then manifest as long-term behavioural changes. However, the extent to which aberrant DNA methylation is involved in the mechanisms of substance use disorder in humans is unclear. We previously demonstrated that cocaine modifies gene transcription, via DNA methylation, throughout the brain and in peripheral blood cells in mice.

**Results:**

We treated human peripheral blood mononuclear cells (PBMCs) from healthy male donors (*n* = 18) in vitro with psychostimulants (amphetamine, cocaine). After treatment, we assessed mRNA levels and enzymatic activities of TETs and DNMTs, conducted genome-wide DNA methylation assays and next-generation sequencing. We found that repeated exposure to psychostimulants decreased mRNA levels and enzymatic activity of TETs and 5-hydroxymethylation levels in PBMCs. These data were in line with observed hyper- and hypomethylation and mRNA expression of marker genes (*IL-10, ATP2B4*). Additionally, we evaluated whether the effects of cocaine on epigenetic editors (DNMTs and TETs) and cytokines interleukin-6 (IL-6) and IL-10 could be reversed by the DNMT inhibitor decitabine.

Indeed, decitabine eliminated cocaine’s effect on the activity of TETs and DNMTs and decreased cytokine levels, whereas cocaine increased IL-6 and decreased IL-10.

**Conclusions:**

Our data suggest that repeated psychostimulant exposure decreases TETs’ enzymatic activity in PBMCs. Co-treatment with decitabine reversed TETs’ levels and modulated immune response after repeated cocaine exposure. Further investigation is needed to clarify if TET could represent a putative biomarker of psychostimulant use and if DNMT inhibition could have therapeutic potential.

**Supplementary Information:**

The online version contains supplementary material available at 10.1186/s13148-022-01303-w.

## Background

Substance use disorder (SUD) is a mental disorder in which there is uncontrolled use of a substance despite harmful consequences [1]. Addiction to psychostimulants (amphetamine, cocaine, etc.) is considered a global health concern with a significant socioeconomic burden to society [2]. At the present time, there is no efficient pharmacotherapy for stimulant-induced addiction, and novel treatment options are greatly needed. A variety of animal studies have implicated a role for epigenetic modifications in the pathogenesis of drug addiction [3–5]. However, it is unknown to what extent aberrant DNA methylation is involved in the mechanisms of SUD in humans. A few studies in humans have reported on the state of DNA methylation in abusers of substances compared with drug-free controls and have provided initial support for findings in animal studies [6–8].

DNA methylation is catalysed by DNA methyltransferases (DNMT1, DNMT3A and -3B) [9, 10]. Several reports have shown that ten-eleven translocation enzymes (TET1-3) participate actively in the DNA demethylation process by adding a hydroxyl group onto the methyl group of 5-methylcytosine (5-mC) to form 5-hydroxymethylcytosine (5-hmC) [11, 12]. Some reports have focused on the role of TET proteins and 5-hmC in the epigenetic regulation of psychostimulant action in animal models [13–16]. The data collected suggest that both DNMT and TET enzymes may function as epigenetic editors in psychostimulant-induced addiction [17, 18].

Our previous study demonstrated that cocaine modified gene transcription, via DNA methylation and demethylation, and that mice had similar changes in epigenetic editors in both the brain and peripheral blood cells [15]. We do not know yet if the mechanisms by which psychostimulants affect epigenetic editors in the PBMCs are also applicable to the human brain. However, PBMCs express proteins related to dopamine transport and signalling, e.g. (DA) receptors, tyrosine hydroxylase (TH) and dopamine transporter (DAT), suggesting that they have the capacity to synthesize, take up, store and release dopamine [19–23]. In addition, in clinical context, PBMCs have an advantage over other tissues in SUD studies because they are readily available from the patients being studied. We speculate that cocaine and amphetamine potentially induce changes through dopamine signalling on epigenetic editors in PBMCs in a similar way that they may act in brain. We also hypothesize that repeated psychostimulant exposure might cause via long-term gene expression changes in epigenetic editors in PBMCs. This would in turn enhance the ability of leukocytes (e.g. monocytes) and cytokines (interleukins) to penetrate the brain and affect neuroplasticity, in addition to a direct effect of psychostimulants in the central nervous system (CNS).

The aim of this study was to investigate whether psychostimulants affect the expression and enzymatic activity of the epigenetic editors and induce aberrant DNA methylation in drug-free human PBMCs. The chosen concentrations of amphetamine and cocaine were based on earlier forensic studies [24, 25] and are expected to be similar to those found in drug users.

Another aim was to find out if the effect of psychostimulants on epigenetic editors and concentrations of cytokines could be reversed after treatment with the DNMT inhibitor decitabine (DAC).

## Methods and materials

**Fig. 1:**
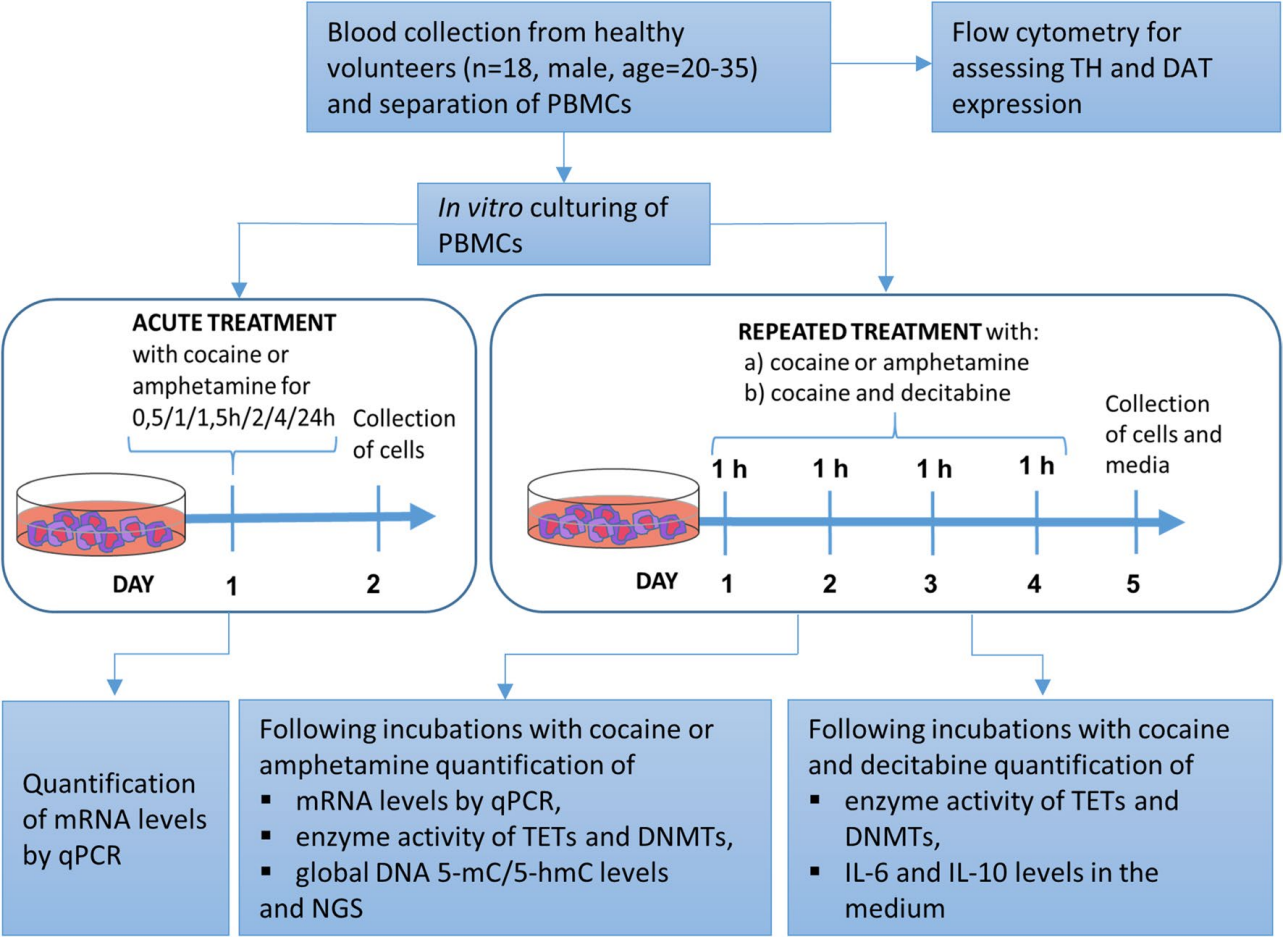
Diagram describing the study design. TH—tyrosine hydroxylase, DAT—dopamine transporter, PBMCs—peripheral blood mononuclear cells, qPCR—quantitative polymerase chain reaction, TET—ten-eleven translocation enzymes, DNMT—DNA methyltransferase, NGS—next-generation sequencing, IL-6/10—interleukin-6/10

The overall design of the study is described in Fig. 1. Detailed information for flow cytometry, Western blot analysis and next-generation sequencing bioinformatics is described in Additional file 1: Supplementary file Methods and Materials section.

### Healthy donors and isolation of PBMCs

Fresh whole blood was collected from healthy male volunteers aged 20–35 years presented in Tartu University Hospital Blood Bank. Written informed consent was obtained from study participants. Participants had to state that they had not used narcotics within the previous year. Human PBMCs were isolated by density centrifugation on the day of blood collection using Ficoll-Paque medium (GE Healthcare, USA) according to manufacturer’s instructions. PBMCs were stored at − 150 °C until use.

### Amphetamine, cocaine and decitabine treatment

PBMCs were cultured in RPMI-1640 medium (Gibco, Thermo Scientific, MA, USA) containing 10% foetal bovine serum (FBS; Invitrogen, CA, USA) on 24-well plates 24 h before the treatments. *Acute treatment*: PBMCs (*n* = 18) were treated in vitro with amphetamine (0.3 µg/ml) or cocaine (3 µg/ml) for 0.5; 1; 1.5; 2; 4 and 24 h. *Repeated treatment:* PBMCs (*n* = 14–18) were treated in vitro with amphetamine (0.3 µg/ml) or cocaine (3 µg/ml) for 1 h/d over a period of 4 consecutive days. *Decitabine* + *Cocaine repeated treatment:* PBMCs (*n* = 14) were treated in vitro with a) cocaine (3 µg/ml), b) decitabine (0.3 µM) or c) simultaneously with cocaine (3 µg/ml) and decitabine (0.3 µM) for 1 h/d over 4 consecutive days. The concentrations of cocaine and amphetamine used in the study have been derived from forensic medical examinations [24, 25]. Based on post-mortem studies, concentrations of cocaine and amphetamine are expected to be even 2 and 3 times higher, respectively, in the brain compared to blood [26]. PBMCs were supplemented with fresh medium after all incubations and grown at 37 °C, in an atmosphere of 95% air and 5% CO_2_. 24 h after last treatment, cells and medium were collected, and cell lysates, RNA, DNA and nuclear extracts were prepared and stored at − 80 °C.

### Flow cytometry

PBMCs from healthy donors (male, age = 20–35, *n* = 6) were thawed, suspended in warm RPMI-1640 medium (Gibco, Thermo Scientific, MA, USA) containing 10% FBS (Invitrogen, CA, USA) and incubated with antibodies for cell surface markers and TH, DAT. Flow cytometry protocol was adapted from a method described by Gopinath and colleagues [27]. For detailed description of flow cytometry, please see Additional file 1: Supplementary file.

### Measuring mRNA levels by qPCR

Total RNA was extracted from the PBMCs using RNeasy Mini Kit (QIAGEN, Hilden, Germany) as previously described [15]. cDNA was synthesized from 375 ng of total RNA using the First Strand cDNA Synthesis Kit (Thermo Scientific, Waltham, MA, USA). qPCR was performed using a QuantStudio 12 K Flex Real-Time PCR System equipped with QuantStudio 12 K Flex Software (Thermo Scientific). The primers (listed in Table 1) were designed using Primer3 with BLAST sequence verification and were synthesized by TAG Copenhagen AS (Denmark). Results were normalized to *B2M* using the comparative C_T_ (${2}^{-\Delta \Delta {C}_{T}}$) method [28].

**Table 1:** qPCR primer sequences

Target genes	Primer sequence
*DNMT1*	Forward: GTTCTTCCTCCTGGAGAATGTT Reverse: GTCTGGGCCACGCCGTACT
*DNMT3A*	Forward: TATTGATGAGCGCACAAGAGAGC Reverse: GGGTGTTCCAGGGTAACATTGAG
*DNMT3B*	Forward: GGCAAGTTCTCCGAGGTCTCTG Reverse: TGGTACATGGCTTTTCGATAGGA
*TET1*	Forward: AATGGAAGCACTGTGGTTTG Reverse: ACATGGAGCTGCTCATCTTG
*TET2*	Forward: GTGAGATCACTCACCCATCG Reverse: CAGCATCATCAGCATCACAG
*TET3*	Forward: GAGGAGCGGTATGGAGAGAA Reverse: AGTAGCTTCTCCTCCAGCGT
*B2M*	Forward: TGCTCGCGCTACTCTCTCT Reverse: TCCATTCTCTGCTGGATGAC
*TH*	Forward: CGGAAGCTGATTGCAGAGAT Reverse: GGGTAGCATAGAGGCCCTTC
*DAT*	Forward: CGAGCCTGCTTGCTGATATT Reverse: ATGGCATCCACTTTCCTGTC
*DRD1*	Forward: AGGGGAATTTGCAGTTCTGT Reverse: AAAAGATGGAGAGGGCCAAT
*DRD2*	Forward: GCAGACCACCACCAACTACC Reverse: CCACTCACCTACCACCTCCA
*DRD3*	Forward: CACTGTCTGCTCCATCTCCA Reverse: GAGGATCCTTTTCCGTCTCC
*DRD4*	Forward: CCTTCTTCGTGGTGCACAT Reverse: AACTCGGCGTTGAAGACAGT
*DRD5*	Forward: GCCTACCAGAGATGGACCAA Reverse: AAAAGGGAGGGGAGAGCATA
*IL-10*	Forward: GCCTAACATGCTTCGAGATC Reverse: TGATGTCTGGGTCTTGGTTC
*ATP2B4*	Forward: AACTCTCAGACTGGAATCATC Reverse: ACCTTTCTTCTTTTTCTCCC

### DNMT and TET activity measurement

Nuclear proteins were extracted from the PBMCs according to the manufacturer’s protocol (Nuclear Extraction kit; ab113474; Abcam, Cambridge, UK). DNMT and TET activity was determined as previously reported [15]. DNMT activity was determined using an Abcam DNMT activity assay kit (ab113467), and a TET Hydroxylase Activity Quantification kit (ab156912) was used to measure TET activity according to the instructions of the manufacturer.

### DNA extraction and Illumina next-generation sequencing (NGS)

DNA from PBMCs (*n* = 4) was extracted using QIAamp DNA Mini kit according to the manufacturer’s protocol (QIAGEN). The NGS library preparation and sequencing was conducted as follows in the Institute of Genomics Core Facility, University of Tartu. Libraries were prepared with TruSeq Methyl Capture EPIC Library Prep kit (Illumina Inc.; San Diego, California, USA), according to manufacturer's instructions and quantified using Illumina-specific KAPA Library Quant Kit (Kapa Biosystems, Wilmington, MA, USA). Sequencing was carried out on an Illumina NextSeq500 System in paired end 2 × 100 bp mode. Sequencing data were demultiplexed using the Illumina Local Run Manager Generate FASTQ Analysis Module v2.0.

### Quantification of global DNA methylation and hydroxymethylation levels

Genomic DNA was extracted from the PBMCs cells as previously described [15]. Global DNA methylation and hydroxymethylation analysis was performed using Global DNA Methylation Assay kit (ab233486; Abcam, Cambridge, UK) and Global DNA Hydroxymethylation Assay kit (ab233487; Abcam, Cambridge, UK) according to the manufacturer’s instructions. The percentage of methylated DNA (5-mC%)/hydroxymethylated DNA (5-hmC%) in total DNA was quantified according to the manufacturer’s protocol and formula.

### Quantitative detection of the IL-6 and IL-10 by ELISA Assay

The secretion of IL-6 and IL-10 in the cellular supernatant (after cocaine and decitabine treatment for 1 h in 4 consecutive days, *n* = 14) was determined using Human IL-6 high-sensitivity ELISA kit (ab46042; Abcam Cambridge, UK) and Human IL-10 high-sensitivity ELISA kit (ab46059; Abcam Cambridge, UK) according to the manufacturer’s protocol. Supernatants were collected 24 h after last cocaine and decitabine treatment.

### Statistical analysis

Levels of mRNA, protein, IL-6, IL-10, 5-mC and 5-hmC, DNMT and TET activity were analysed using one-way ANOVAs with Bonferroni’s post hoc test. GraphPad Prism software (San Diego, CA, USA) was used for statistical analyses, and significance levels were set to *p* < 0.05.

## Results

### Expression of TH and DAT on monocytes and T cells

**Fig. 2:**
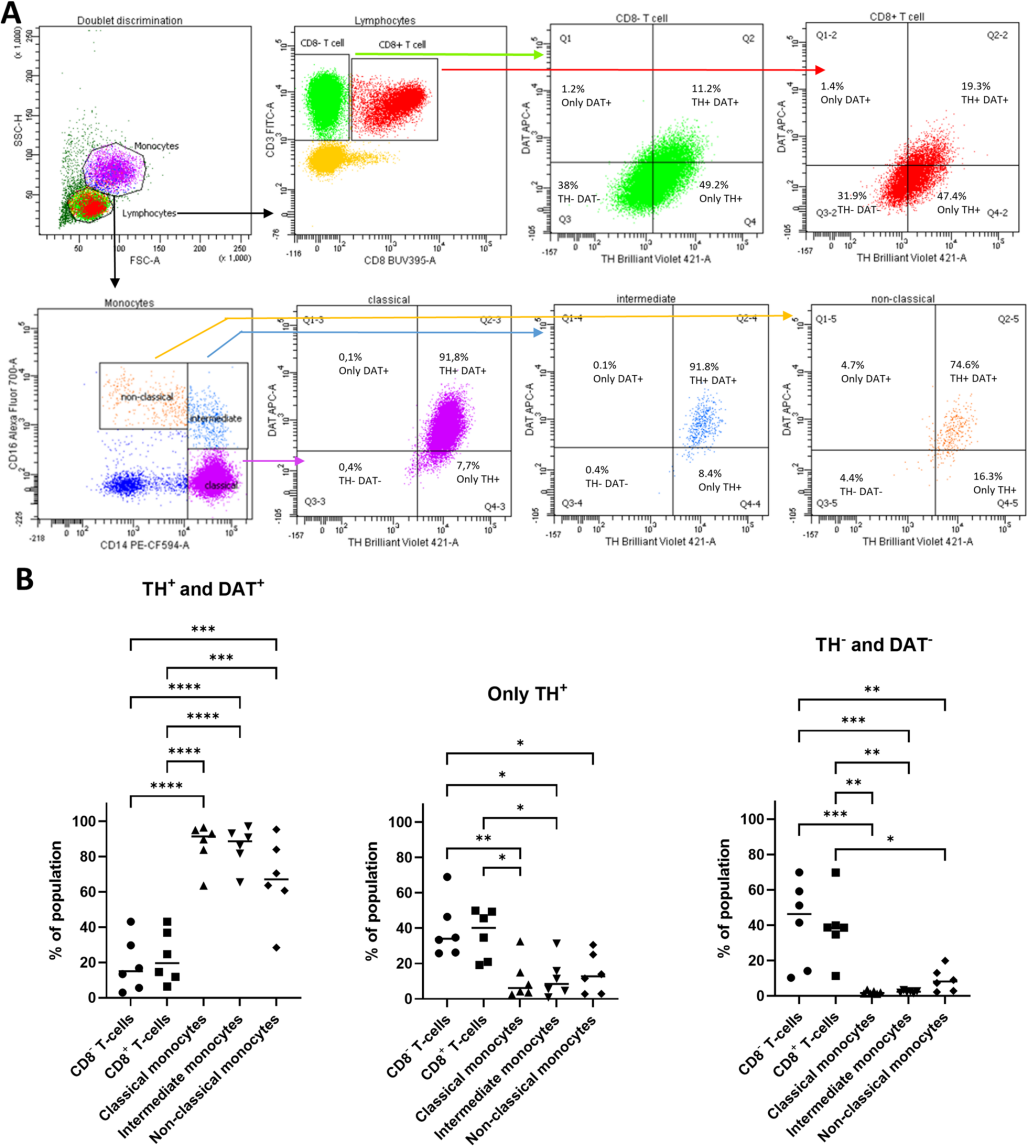
Expression of tyrosine hydroxylase (TH) and dopamine transporter (DAT) in peripheral blood mononuclear cells (PBMCs). Representative plots showing the gating strategy for separation of lymphocyte and monocyte subsets and the expression of TH and DAT in naïve PBMCs from a donor (**A**). Lymphocytes and monocytes were determined by side-scatter height (SSC-H) and forward-scatter area (FSC-A). T cells were stained with surface markers CD8 and CD3 and divided into CD8 + and CD8- T cells. Classical, intermediate and non-classical monocytes were defined based on the expression of CD16 and CD14. The percentage of TH^+^ and DAT^+^ double-positive cells was higher in monocytes subsets than in T cell subsets (**B**). In addition, T cells subsets had a higher percent of cells expressing only TH and a higher percentage of populations were TH^−^ and DAT^−^ double negative than in monocyte subsets. One-way ANOVA followed by Bonferroni post hoc test; *p < 0.05, **p < 0.01, ***p < 0.001, ****p < 0.0001, *n* = 6

In a flow cytometry study, we found that T cells and monocytes expressed TH and DAT (Fig. 2), whereas the percentage of TH^+^ and DAT^+^ double-positive cells was higher in monocytes than in T cells (one-way ANOVA main effect of group: *F*_*(4,25)*_ = 26.69, *p* < 0.0001; followed by Bonferroni post hoc test; *p* < 0.001: CD8^−^ T cells vs non-classical monocytes; CD8^+^ T cells vs non-classical monocytes; *p* < 0.0001: CD8^−^ T cells vs classical/intermediate monocytes; CD8^+^ T cells vs classical/intermediate monocytes). More T cells expressed only TH than monocytes (one-way ANOVA main effect of group: *F*_(*4,25*)_ = 7.052, *p* = 0.0006; followed by Bonferroni post hoc test; *p* < 0.05: CD8^−^ T cells vs intermediate/non-classical monocytes; CD8^+^ T cells vs classical/intermediate monocytes; *p* < 0.01: CD8^−^ T cells vs classical monocytes). The percentage of TH and DAT double-negative monocytes was low compared to T cells (one-way ANOVA main effect of group: *F*_(*4,25*)_ = 11.82, *p* < 0.0001; followed by Bonferroni post hoc test, *p* < 0.05: CD8^+^ T cells vs non-classical monocytes; *p* < 0.01 CD8^−^ T cells vs non-classical monocytes; CD8^+^ T cells vs classical/intermediate monocytes; *p* < 0.001: CD8^−^ T cells vs classical/intermediate monocytes). No differences were detected in the percentage of TH^+^DAT^+^, TH^+^ or TH^−^DAT^−^ cells within the monocyte or T cell subsets.

### Changes in the expression levels of DNMTs, TETs, DAT, TH and DA receptors in response to acute and repeated psychostimulant exposure

qPCR data showed that amphetamine acute exposure increased *DNMT3A* mRNA levels at 24 h after acute amphetamine treatment (Additional file 1: Figures S3, S4).

**Fig. 3:**
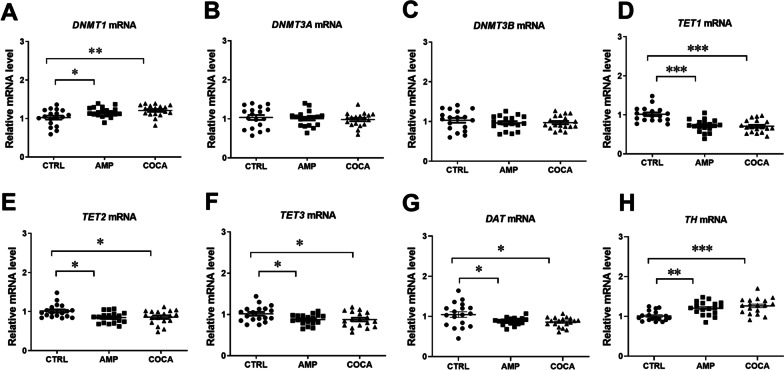
Changes in the mRNA levels of *DNMT*s (**A**–**C**), *TET*s (**D**–**F**), *DAT* (**G**) and *TH* (**H**) in response to repeated amphetamine or cocaine exposure in vitro in human peripheral blood mononuclear cells (PBMCs). One-way ANOVA, followed by Bonferroni post hoc test; *p < 0.05, **p < 0.01, ***p < 0.001, *n* = 18 in all groups. CTRL = control, AMP = amphetamine, COCA = cocaine. Error bars indicate SEM

Repeated psychostimulant treatment upregulated mRNA levels of *DNMT1* but did not affect *DNMT3A* and *DNMT3B* mRNA levels in PBMCs (Fig. 3A–C; *DNMT1,* one-way ANOVA main effect of group: *F*_(2,51)_ = 6.035, *p* = 0.0044; followed by Bonferroni post hoc test; *p* < 0.05, *p* < 0.01 compared with the control group; *n* = 18). We also found that repeated psychostimulant exposure reduced *TET1-3* mRNA levels (Fig. 3D–F; *TET1,* one-way ANOVA main effect of group: *F*_(2,51)_ = 18.93, *p* < 0.0001; *TET2, F*_(2,51)_ = 5.831, *p* = 0.0052; *TET3, F*_(2,51)_ = 4.188, *p* = 0.0207*;* followed by Bonferroni post hoc test; *p* < 0.05, *p* < 0.001 compared with the control group;* n* = 18). Furthermore, repeated psychostimulant treatment significantly decreased *DAT* and increased *TH* mRNA levels (Fig. 3G–H; *DAT,* one-way ANOVA main effect of group: *F*_(2,51)_ = 5.054, *p* = 0.0099; *TH, F*_(2,51)_ = 11.57, *p* < 0.0001; followed by Bonferroni post hoc test; *p* < 0.05, *p* < 0.01, *p* < 0.001 compared with the control group;* n* = 18). In this study, we also assessed repeated amphetamine and cocaine treatment effects on dopamine receptors (*DRD1-5*) mRNA levels, but there were no statistically significant changes (Additional file 1: Figure S5). Western blotting did not reveal any significant changes in DNMT1 and TET1 protein levels in PBMCs after treatments with psychostimulants (Additional file 1: Figure S6).

### Changes in DNMT and TET enzyme activity in response to repeated psychostimulants exposure

**Fig. 4:**
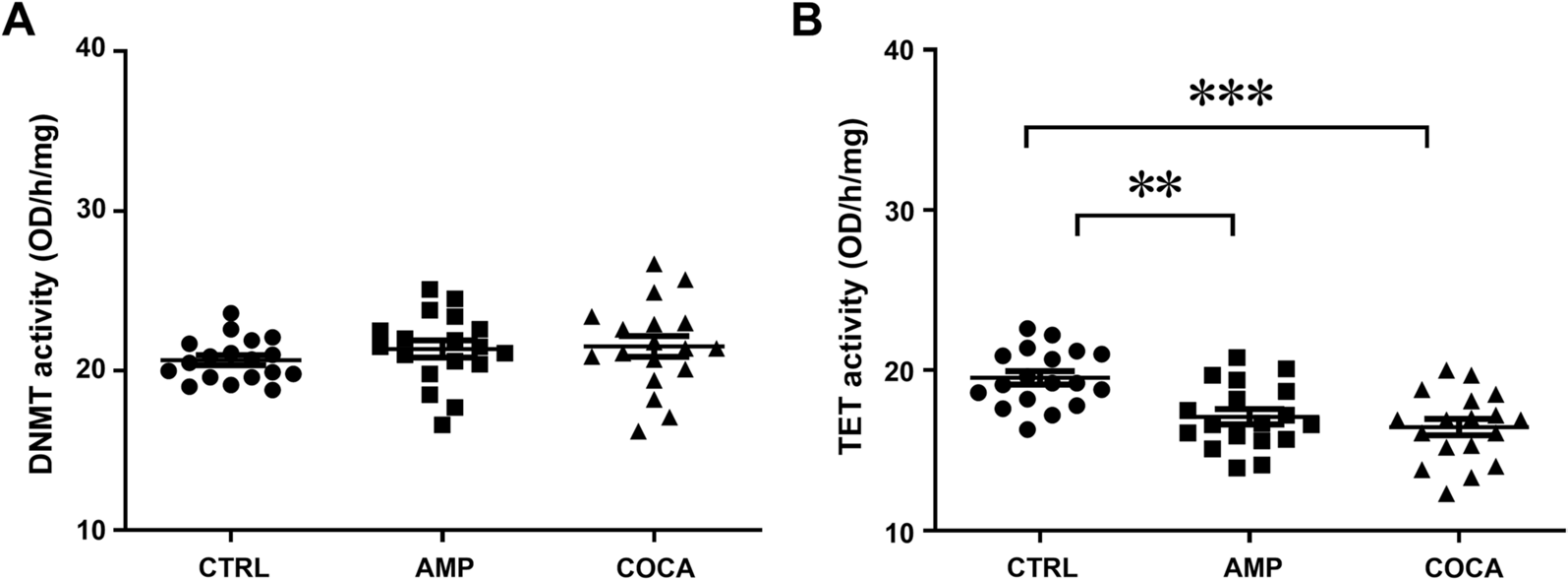
Changes in DNMT (**A**) and TET (**B**) enzyme activity levels in response to repeated amphetamine or cocaine exposure in human PBMCs. One-way ANOVA, followed by Bonferroni post hoc test; **p < 0.01, ***p < 0.001, *n* = 18 in all groups. CTRL = control, AMP = amphetamine, COCA = cocaine; OD = optical density. Error bars indicate SEM

We measured the activities of DNMT and TET enzymes in PBMCs. There was no statistically significant change in DNMT activity after repeated psychostimulant exposure (Fig. 4A*,* one-way ANOVA main effect of group: *F*_(2,51)_ = 0.7851, *p* = 0.4615; *n* = 18). However, repeated psychostimulant treatment significantly decreased TET activity in PBMCs (Fig. 4B, one-way ANOVA main effect of group: *F*_(2,51)_ = 11.87, *p* < 0.001; followed by Bonferroni post hoc test; *p* < 0.01, *p* < 0.001 compared with the control group;* n* = 18).

### Next-generation sequencing

We studied the genome-wide DNA methylation profiles in PBMCs (*n* = 4) after repeated treatments with psychostimulants. Our data showed that in the amphetamine group, 76.21% of cytosine-phosphate-guanine (CpG) sites were hypermethylated, while 23.78% of CpG sites were hypomethylated compared with the control group. In the cocaine group, we found that 76.47% of CpG sites were hypermethylated, while 23.52% of CpG sites were hypomethylated. At the gene level, in the amphetamine group, 52.62% of genes were hypermethylated, while 47.38% genes were hypomethylated compared with the control group. In the repeated cocaine treatment group, we found that 54.04% of genes were hypermethylated, while 45.96% of genes were hypomethylated, respectively. Gene ontology (GO) analysis revealed that both repeated amphetamine and cocaine treatment tended to hypermethylate genes involved in biological processes that included immune responses, inflammatory and cytokine responses, leukocyte cell–cell adhesion, locomotion and the regulation of transcription (Additional file 2: Tables S1, S3), and mostly hypomethylated genes involved in biological processes that included nervous system process, receptor signalling pathway, immune responses and cytokine responses (Additional file 2: Tables S2, S4).

**Fig. 5:**
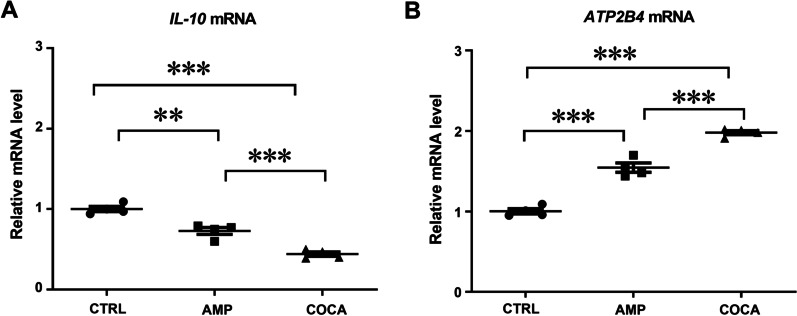
Changes in the mRNA levels of *IL-10* (**A**) and *ATP2B4* (**B**) in response to repeated amphetamine or cocaine exposure in human PBMCs. One-way ANOVA, followed by Bonferroni post hoc test; **p < 0.01, ***p < 0.001, *n* = 4 in all groups. CTRL = control, AMP = amphetamine, COCA = cocaine. Error bars indicate SEM

As we found that repeated amphetamine and cocaine treatment significantly hypermethylated (*p* < 0.001) interleukin 10 gene (*IL-10,* listed in the top gene list for both amphetamine and cocaine, Additional file 2: Tables S1 and S3) and significantly hypomethylated (*p* < 0.001) the ATPase Plasma Membrane Ca^2+^ Transporting 4 gene (*ATP2B4,* listed in the top gene list for both amphetamine and cocaine, Additional file 2: Tables S2 and S4), we measured *IL-10* and *ATP2B4* mRNA levels after repeated psychostimulant treatment in PBMCs. Our qPCR data showed that repeated psychostimulant treatment significantly decreased *IL-10* and increased *ATP2B4* mRNA levels (Fig. 5A, one-way ANOVA main effect of group: *F*_(2,9)_ = 64.64, *p* < 0.001; followed by Bonferroni post hoc test; *p* < 0.001 compared with the control group; *p* < 0.001 amphetamine *vs* cocaine; Fig. 5B, *F*_(2,9)_ = 145.9, *p* < 0.001; followed by Bonferroni post hoc test; *p* < 0.001 compared with the control group; *p* < 0.001 amphetamine *vs* cocaine; *n* = 4).

### Changes in global 5-hmC levels in response to repeated psychostimulants exposure

**Fig. 6:**
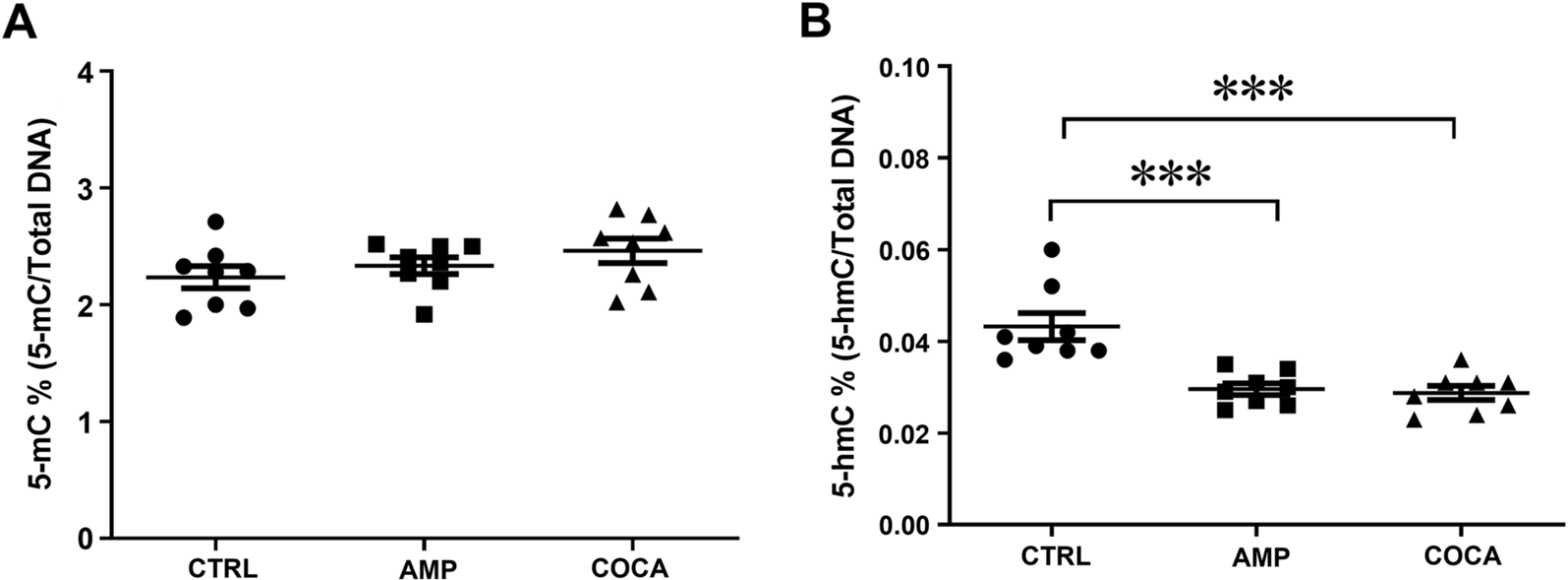
Global DNA methylation (**A**) and hydroxymethylation (**B**) levels in human PBMCs in response to repeated amphetamine or cocaine exposure. One-way ANOVA, followed by Bonferroni post hoc test; ***p < 0.001, *n* = 8 in all groups. CTRL = control, AMP = amphetamine, COCA = cocaine. Error bars indicate SEM

The genome-wide DNA methylation analysis did not distinguish between methyl and hydroxymethyl groups. Therefore, we analysed global 5-mC and 5-hmC levels in PBMCs in response to repeated amphetamine and cocaine exposure. We did not detect any statistically significant change in global 5-mC levels after repeated psychostimulant treatment (Fig. 6A). Regarding 5-hmC levels, our data showed that repeated amphetamine or cocaine treatment decreased global 5-hmC levels (Fig. 6B, one-way ANOVA main effect of group: *F*_(2,21)_ = 15.58, *p* < 0.001; followed by Bonferroni post hoc test; *p* < 0.001 compared with the control group; *n* = 8).

### Changes in DNMT and TET enzyme activities, IL-6 and IL-10 levels following decitabine and cocaine exposure

**Fig. 7:**
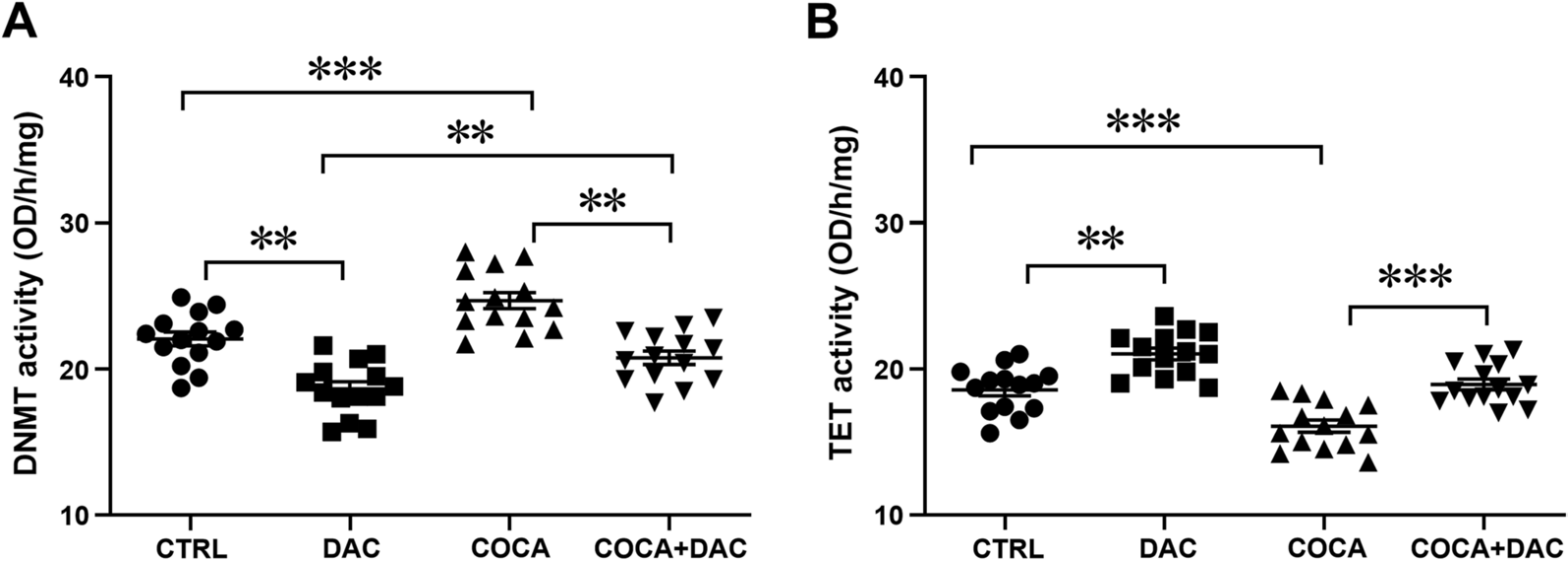
Changes in DNMT (**A**) and TET (**B**) enzyme activity levels in response to repeated decitabine, cocaine and decitabine + cocaine exposure in human PBMCs. One-way ANOVA, followed by Bonferroni post hoc test; **p < 0.01, ***p < 0.001, *n* = 14 in all groups. CTRL = control, DAC = decitabine, COCA = cocaine. Error bars indicate SEM

To find an optimal dose for DNMT inhibitor DAC, we conducted a pilot study with different doses of DAC while monitoring PBMC viability (colorimetric MTT test, data not shown). Based on these data, we selected a dose of 0.3 µM DAC for subsequent studies. DNMT activity data showed that repeated DAC treatment significantly decreased and cocaine treatment increased DNMT activity in PBMCs, whereas repeated cocaine and DAC co-treatment normalized DNMT activity (Fig. 7A, one-way ANOVA main effect of group: *F*_(3,52)_ = 25.74, *p* < 0.0001; followed by Bonferroni post hoc test; *p* < 0.01, *p* < 0.001 compared with the control group; *p* < 0.01 cocaine and DAC *vs* cocaine + DAC co-treatment;* n* = 14). Repeated DAC treatment significantly increased and cocaine treatment decreased TET activity in PBMCs, whereas repeated cocaine and DAC co-treatment normalized TET activity (Fig. 7B, one-way ANOVA main effect of group: *F*_(3,52)_ = 25.23, *p* < 0.0001; followed by Bonferroni post hoc test; *p* < 0.01, *p* < 0.001 compared with the control group; *p* < 0.001 cocaine *vs* cocaine + DAC co-treatment;* n* = 14).

**Fig. 8:**
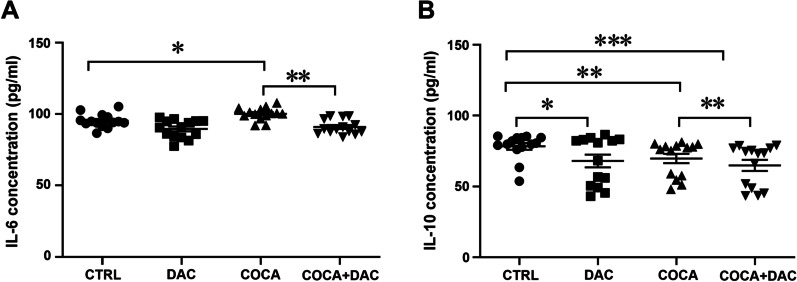
IL-6 (**A**) and IL-10 (**B**) concentration levels in response to repeated decitabine, cocaine and decitabine + cocaine exposure in human PBMCs cellular supernatant. One-way ANOVA, followed by Bonferroni post hoc test; *p < 0.05, **p < 0.01, ***p < 0.001, *n* = 14 in all groups. CTRL = control, DAC = decitabine, COCA = cocaine. Error bars indicate SEM

Our next aim was to measure IL-6 and IL-10 levels in PBMC supernatant following repeated DAC and cocaine treatment. As illustrated in Fig. 8, repeated cocaine treatment significantly increased IL-6 levels. Interestingly, repeated cocaine and DAC co-treatment also slightly decreased IL-6 levels (Fig. 8A, one-way ANOVA main effect of group: *F*_(3,52)_ = 11.69, *p* < 0.0001; followed by Bonferroni post hoc test; *p* < 0.05 compared with the control group; *p* < 0.01 cocaine *vs* cocaine + DAC co-treatment;* n* = 14). Regarding IL-10 levels in PBMC supernatant, our data showed that repeated DAC or cocaine treatment and also repeated cocaine and DAC co-treatment significantly decreased IL-10 levels (Fig. 8B, one-way ANOVA main effect of group: *F*_(3,52)_ = 11.72, *p* = 0.0004; followed by Bonferroni post hoc test; *p* < 0.05, *p* < 0.01, *p* < 0.001 compared with the control group; *p* < 0.01 cocaine *vs* cocaine + DAC co-treatment;* n* = 14).

## Discussion

As epigenetic editors cannot be assessed in the brains of living humans, we were compelled to look for an alternative model to evaluate the effects of psychostimulants in humans. In the present work, we mimic the effects of acute and repeated administration of psychostimulants using isolated human PBMCs.

The first aim was to elucidate the effect of psychostimulants on DNA methylation and epigenetic editors conducting DNA methylation or demethylation (DNMTs and TETs) in human PBMCs. Our results confirmed that human PBMCs express TH and DAT, necessary for synthesis and transport of dopamine. Moreover, the results revealed that repeated exposure of amphetamine and cocaine decreased mRNA levels and enzymatic activity of TETs and 5-hmC levels in PBMCs. We did not find any evidence of acute exposure of amphetamine or cocaine on mRNA levels of TETs; however, the mRNA levels of DNMT3A were increased. Falling in line with a decrease in global 5-hmC levels after psychostimulant exposure, next-generation sequencing showed that after repeated incubations with amphetamine and cocaine hypermethylation predominated on cytosine-phosphate-guanine (CpG) sites, however, when analysing changes in DNA methylation at the gene level, both amphetamine and cocaine exposure cause relatively equal hyper- and hypomethylation. Two marker genes which were found in top lists for hypermethylated genes (*IL-10*) and hypomethylated genes (*ATP2B4*) after repeated incubations with cocaine and amphetamine were chosen for further analysis. qPCR results confirmed that hypermethylation of *IL-10* gene resulted in decrease in its mRNA levels and hypomethylation of *ATP2B4* resulted in an increase in its mRNA levels after repeated incubations with psychostimulants.

The second aim was to elucidate if the effect of repeated exposure to cocaine causing aberrant DNA methylation and levels of cytokines could be reversed by using a DNMT inhibitor (DAC) in PBMCs. Indeed, DAC eliminated cocaine’s effect on the activity of TETs and DNMTs. In addition, while treatment with only cocaine increased IL-6 levels and decreased IL-10 levels, co-treatment with cocaine and DAC decreased cytokines’ IL-6 and IL-10 levels.

Our flow cytometry and qPCR results confirm that PBMCs express DAT, TH and DA receptors. We found that monocytes express both DAT and TH, whereas there are more T cells that express only TH compared to monocytes, suggesting that different PBMC populations may respond differently to psychostimulant exposure. qPCR results indicate that acute and repeated exposure of psychostimulants did not affect mRNA levels of DA receptors, whereas repeated administration of psychostimulants decreased DAT and increased TH expression in PBMCs. These results support previous studies suggesting that immune signalling critically regulates homeostasis of the nervous system [29].

Cells rely on enzymatic activities of DNMTs and TETs together to maintain genomic methylation homeostasis, and imbalances in methylation homeostasis contribute to various diseases [30]. Following acute incubations with psychostimulants, we did not detect any changes in gene expression of the epigenetic editors (except *DNMT3A* mRNA levels 24 h following acute amphetamine treatment) in PBMCs. However, repeated exposure to psychostimulants significantly decreased *TET1-3* expression and was associated with a decrease in enzymatic activity of TETs. We also found a significant increase in *DNMT1* mRNA levels, but no changes of *DNMT3A* and *-3B* expression after psychostimulant exposure. We speculate that an adaptation response is responsible for changes in the epigenetic editors in PBMCs following an environmental factor (e.g. psychostimulant) exposure. The mechanism of these changes has not been fully elucidated but may be related to activation of the peripheral dopaminergic system.

Several reports have previously shown in rodent models of addiction that the most prominent enzyme involved in DNA methylation in the brain is DNMT3A and that the expression of DNMT1 and DNMT3B is relatively low [4, 5, 31]*.* In a previous report, we found that there were similarities between both DNMT and TET expression and enzymatic activities between the brain and peripheral blood cells [15]. However, in the current study, the primary effect was a decrease in the expression and enzymatic activity of TETs, suggesting that in humans there may be a difference between the expression of epigenetic editors after repeated psychostimulant exposure in PBMCs. This epigenetic difference between mice and humans may be due to differences in experimental models but may also be species-specific.

A decrease in TETs’ activity as a potential biomarker for psychostimulant use could be investigated in the future. Data from our preclinical study suggest that psychostimulants affect the epigenetic editors in a similar manner in the brain and in peripheral blood cells [15]. However, we do not know if this is the case in humans. Our current study limitations are in vitro and not in vivo study design as well as not assessing any human-origin brain tissue to validate if similar changes in the activity of TETs are occurring in the brain.

We performed NGS to evaluate the effect of psychostimulants on the genome-wide DNA methylation profiles in PBMCs. The bioinformatic analyses indicated that, in general, amphetamine and cocaine have similar effects on genome-wide DNA methylation. When analysing changes in DNA methylation at the gene level, we found that both amphetamine and cocaine exposure cause relatively equal hyper- and hypomethylation. However, in the analysis performed at the level of CpG sites (including non-coding regions of the genes), hypermethylation predominated. We speculate that some of these non-coding regions may function as a transcriptional enhancer. These transcriptional enhancers bind chromatin-modulating factors, interact with distal promoters through DNA loops, and demonstrate a unique pattern of DNA methylation [32]. The enhancer sites that contain CG dinucleotides become demethylated upon their activation, concomitant with transcription factor binding [33, 34].

Our GO analysis showed that after repeated psychostimulant treatment, both hyper- and hypomethylated genes were involved in biological processes, such as immune and cytokine responses. We selected two marker genes (*IL-10 and ATP2B4*) for further analysis as their methylation pattern had changed in PBMCs after repeated exposure to psychostimulants. The psychostimulant treatment significantly hypermethylated *IL-10* and hypomethylated *ATP2B4,* and these changes correlated with altered mRNA expression. In humans, cytokine IL-10 is mainly produced by lymphocytes, monocytes, macrophages and dendritic cells [35]. In the CNS, microglia and astroglia are potential sources of IL-10 [36, 37]. IL-10 regulates a variety of immune cells to limit and stop the inflammatory response and thus plays an important role in autoimmune diseases, inflammatory diseases and cancer [38]. Moreira and colleagues [39] demonstrated that subjects who reported cocaine use had a decrease in IL-10 serum levels. Similarly, reduced plasma levels of IL-10 were demonstrated among cocaine dependents [40]. Among the hypomethylated genes, we analysed *ATP2B4*, which is expressed at high levels in numerous tissues and cell types, including the brain, heart and spermatozoa [41]. Following repeated psychostimulant treatment, *ATP2B4*, among other genes, showed different expression in human blood and in rhesus macaques brain [42, 43]. Therefore, our results describing alterations in marker gene expression and methylation after treatment with cocaine and amphetamine are supported by earlier findings and indicate that our in vitro model was able to mimic in vivo effect of psychostimulants.

To analyse the balance between DNA methylation and demethylation, we assessed global 5-mC and 5-hmC levels in PBMCs. Our results suggest that the downregulation of TET activity leads to reduced 5-mC conversion to 5-hmC, resulting in promoter hypermethylation and transcriptional repression, respectively. These genome-wide 5-mC and 5-hmC findings are in line with previous reports [44–46]. However, Feng and colleagues [13] demonstrated that TET1 downregulation, after repeated cocaine treatment, increased the enrichment of 5-hmC at the putative enhancers and gene bodies and correlated with increased expression of these genes in mice. These data indicate that a decrease in the expression of TETs may result in differential global and site-specific DNA methylation levels. The results of both NGS and the global 5-mC and 5-hmC study also suggest that a new DNA methylation and demethylation balance may occur following psychostimulant treatment in PBMCs.

Our data demonstrated that repeated treatment with psychostimulants significantly reduced the enzymatic activity of TETs. Therefore, our next goal was to investigate whether this effect of cocaine could be reversed. As a selective TET activator is not known and a recent study showed that the DNMT inhibitor DAC also causes an increase in 5-hmC levels in human leukaemia cells [47], we chose DAC for our subsequent studies. DAC is a cytidine deoxynucleoside analogue that is incorporated into DNA strands upon replication. DNMTs are bound to DAC irreversibly and cannot disengage, causing DNMT inhibition at low doses [48, 49]. DAC decreased DNA hypermethylation of CG-rich regions in promoters of genes and restored transcriptional activity of those loci. Interestingly, in addition to the decrease in DNMT activity, we found that repeated DAC exposure significantly increased basal activity of TET and normalized the enzymatic activity of TET after repeated cocaine exposure in PBMCs. The exact molecular mechanism by which DAC increases TET activity is not conclusive; however, others have suggested that the increased abundance of 5-hmC after DAC treatment may be due to increased binding of TET at hemi-methylated dyads [47]. Taken together, our results are consistent with previous studies showing that DAC reduces 5-mC but increases 5-hmC levels in mitotic cells.

To further explore the relationship between cocaine exposure and immune response, we evaluated whether cocaine and DAC treatment affected cytokines, which play a critical role in the communication between the immune system and the CNS [50]. IL-6 is the main pro-inflammatory cytokine activated in the innate immune responses. Studies have shown that IL-6 is also involved in the regulation of brain neuroplasticity [51]. The major biologic function of IL-10 is to limit and stop the inflammatory response, although some studies have shown a role for IL-10 in restoring neuroplasticity [52, 53]. Here, we found that IL-6 and IL-10 concentrations were in line with previous results that showed increased plasma pro-inflammatory molecules (mainly IL-6 and TNF-a) and decreased anti-inflammatory molecules (IL-10), in cocaine users compared to healthy controls [39, 54, 55]. Expression and secretion of IL-6 and IL-10 are thought to be regulated by epigenetic mechanisms. Tang and colleagues [56] demonstrated that the up-regulation of IL-6 was modulated by promoter demethylation in human lung epithelial cells and that DAC administration enhanced IL-6 promoter activity in a dose-dependent manner. Accumulating data suggest that epigenetic modifications play an important role in the regulation of IL-10 [38, 57]. Previous studies have shown that hypomethylation of the IL-10 promoter leads to higher IL-10 expression in PBMCs [58]. We found that cocaine hypermethylated the *IL-10* gene resulting in decreased IL-10 mRNA levels and concentration in the cell culture medium, indicating that DNA methylation may be a key regulatory mechanism for IL-10 expression. DAC treatment significantly reduced IL-10 basal concentrations and further reduced cocaine-induced decreases in IL-10 concentration in the cell culture medium. Thus, our results demonstrate that psychostimulants alter immune signalling and may induce neuroinflammation.

Even though DAC is a medicine approved by regulatory authorities, it is a chemotherapy drug with severe side effects [59]. Current in vitro study is insufficient to make conclusions about the therapeutic potential in a more complicated system at the organism level. However, our study indicates a proof to concept that DNMT inhibition reverses the cocaine-induced changes on the DNMTs and TETs enzymatic activities and modulates the immune response in the PBMCs.

In addition to the possible indirect effect of cytokines on brain functions, monocytes whose function peripherally has been altered by psychostimulant exposure might penetrate the brain and affect neuroplasticity by altered cytokine production. Previous findings suggest that psychostimulants may induce dysfunction of the blood–brain barrier (BBB) by inducing neuroinflammatory pathways, increasing enzyme activation related to BBB remodelling and alterations in tight junction protein expression [60]. A recent study by Niu and colleagues [61] showed increased penetration of monocytes into the brain when examining post-mortem tissue from cocaine users.

Regarding the non-CNS-related effects of psychostimulants, data suggest that psychostimulants may modulate the immune system [40]. Studies have observed a higher risk of contracting or transmitting infectious diseases among cocaine users compared to non-users [54, 62–64]. Some studies support the hypothesis that cocaine exposure may lead to long-lasting pathophysiological changes in the immune system that worsen HIV outcomes [65].

## Conclusions

Our study identified that repeated treatments with either cocaine or amphetamine decreased the enzymatic activity of TETs in PBMCs. Further investigation is needed to clarify if TET could be a putative biomarker for users of repeated psychostimulants, contributing to the development of SUD diagnosis. Even though data from our study in mice suggest that alterations in the activities of epigenetic editors after cocaine treatment are similar between peripheral blood cells and the CNS [15], it is not yet known whether we can extrapolate the activity of epigenetic editors in human PBMCs to the CNS. Previous reports indicate that DNA modification pattern profiles can vary widely between tissues and cell types [66], and caution should therefore be taken in extrapolating results from peripheral tissues to the brain. However, we hypothesize that the activity of epigenetic editors varies less between different tissues, and that TET activity can be useful as a biomarker for various conditions associated with SUD.

Additionally, our results suggest that DNMT inhibition by DAC reverses the effects of psychostimulants on the enzymatic activities of TETs and DNMTs and modulates the immune response in PBMCs. Further investigation is needed to clarify if DNMT inhibitors could have therapeutic potential.

## Supplementary Information

**Additional file 1: Table S1.** Configuration details of LSR Fortessa optical detectors. **Figure S1.** Gating strategy for defining T-lymphocyte and monocyte subsets. **Figure S2.** Staining of tyrosine hydroxylase (TH) and dopamine transporter (DAT) in negative controls. **Figure S3.** Changes in the mRNA levels of DNMTs (A–C) and TET1-3 (D–F), in response to acute amphetamine exposure in vitro in human peripheral blood mononuclear cells (PBMCs). One-way ANOVA, followed by Bonferroni post-hoc test; *p < 0.05, n = 18 in all groups. CTRL = control. Error bars indicate SEM. **Figure S4.** Changes in the mRNA levels of DNMTs (A–C) and TET1-3 (D–F) in response to acute cocaine exposure in vitro in human PBMCs. One-way ANOVA, followed by Bonferroni post-hoc test; p > 0.05, n = 18 in all groups. CTRL = control. Error bars indicate SEM. **Figure S5.** Changes in the mRNA levels of DRD1-DRD5 (A–E) in response to repeated amphetamine and cocaine exposure in human PBMCs. One-way ANOVA, followed by Bonferroni post-hoc test; p > 0.05, n = 8 in all groups. CTRL = control, AMP = amphetamine, COCA = cocaine. Error bars indicate SEM. **Figure S6.** Changes in DNMT1 (A) and TET1 (B) protein levels in response to repeated amphetamine and cocaine exposure in human PBMCs. One-way ANOVA, followed by Bonferroni post-hoc test; p > 0.05, n = 8 in all groups. Error bars indicate SEM. Full western blot images for quantification of protein levels of DNMT1 (C) and TET1 (D). Samples from four donors are represented on the images. CTRL = control, AMP = amphetamine, COCA = cocaine, OD = optical density, β-act = β-actin, MW = molecular weight.

**Additional file 2: Table S1.** Control vs Amphetamine hypermethylated genes (p < 0.001). **Table S2.** Control vs Amphetamine hypomethylated genes (p < 0.001). **Table S3.** Control vs Cocaine hypermethylated genes (p < 0.001). **Table S4.** Control vs Cocaine hypomethylated genes (p < 0.001).

## Data Availability

All data generated or analysed during this study are included in this published article and its supplementary information files. Additional data are available from the corresponding author on reasonable request.

## Abbreviations

AMP: Amphetamine
ATP2B4: ATPase Plasma Membrane Ca^2+^ Transporting 4 gene
BBB: Blood-brain barrier
COCA: Cocaine
CNS: Central nervous system
CpG: Cytosine-phosphate-guanine
CTRL: Control
DA: Dopamine
DAC: Decitabine
DAT: Dopamine transporter
DNMT: DNA methyltransferase
DRD: Dopamine receptor
GO: Gene ontology
IL-6: Interleukin 6
IL-10: Interleukin 10
5-mC: 5-Methylcytosine
5-hmC: 5-Hydroxymethylcytosine
PBMC: Peripheral blood mononuclear cells
qPCR: Quantitative polymerase chain reaction
SUD: Substance use disorder
TET: Ten-eleven translocation enzymes
TH: Tyrosine hydroxylase
