# Supplementary material for: Psychostimulant-induced aberrant DNA methylation in an in vitro model of human peripheral blood mononuclear cells
Source: Clin Epigenetics. 2022 Jul 16;14:89. doi: 10.1186/s13148-022-01303-w (PMC9288712; doi:10.1186/s13148-022-01303-w)
Supplement: Supplementary file 1 — Additional file 1: Table S1. Configuration details of LSR Fortessa optical detectors. Figure S1. Gating strategy for defining T-lymphocyte and monocyte subsets. Figure S2. Staining of tyrosine hydroxylase (TH) and dopamine transporter (DAT) in negative controls. Figure S3. Changes in the mRNA levels of DNMTs (A–C) and TET1-3 (D–F), in response to acute amphetamine exposure in vitro in human peripheral blood mononuclear cells (PBMCs). One-way ANOVA, followed by Bonferroni post-hoc test; *p < 0.05, n = 18 in all groups. CTRL = control. Error bars indicate SEM. Figure S4. Changes in the mRNA levels of DNMTs (A–C) and TET1-3 (D–F) in response to acute cocaine exposure in vitro in human PBMCs. One-way ANOVA, followed by Bonferroni post-hoc test; p > 0.05, n = 18 in all groups. CTRL = control. Error bars indicate SEM. Figure S5. Changes in the mRNA levels of DRD1-DRD5 (A–E) in response to repeated amphetamine and cocaine exposure in human PBMCs. One-way ANOVA, followed by Bonferroni post-hoc test; p > 0.05, n = 8 in all groups. CTRL = control, AMP = amphetamine, COCA = cocaine. Error bars indicate SEM. Figure S6. Changes in DNMT1 (A) and TET1 (B) protein levels in response to repeated amphetamine and cocaine exposure in human PBMCs. One-way ANOVA, followed by Bonferroni post-hoc test; p > 0.05, n = 8 in all groups. Error bars indicate SEM. Full western blot images for quantification of protein levels of DNMT1 (C) and TET1 (D). Samples from four donors are represented on the images. CTRL = control, AMP = amphetamine, COCA = cocaine, OD = optical density, β-act = β-actin, MW = molecular weight. [file 13148_2022_1303_MOESM1_ESM.docx]

**Additional file 1**

**Methods and Materials**

**Flow cytometry**

Briefly, naïve PBMCs were stained with cell surface markers mouse anti CD3 1:100 (300454, Biolegend, CA, USA), mouse anti CD8 1:200 (563795, BD Biosciences, CA, USA), mouse anti CD14 1:500 (301851, Biolegend, CA, USA) and mouse anti CD16 1:500 (392406, Biolegend, CA, USA) in flow cytometry buffer (PBS (pH 7.2), 2 mM EDTA, 0.5% BSA) for 30 min at +4°C following an incubation with IC Fixation Buffer (88-8824-00, eBioscience™ Intracellular Fixation & Permeabilization Buffer Set, Invitrogen, CA). Cells were then stained for 30 min at room temperature in dark with primary antibodies: rat anti dopamine transporter (DAT) 1:100 (MAB369, Merck Millipore, MA, USA) and rabbit anti tyrosine hydroxylase (TH) 1:100 (AB152, Merck Millipore, MA, USA) following an incubation with secondary antibodies anti-rat IgG-APC (551019, BD Biosciences, CA, USA) 1:40; anti-rabbit IgG-B421 (565014, BD Biosciences, CA, USA) 1:40 in permeabilization buffer (eBioscience™ Intracellular Fixation & Permeabilization Buffer Set, Invitrogen, CA, USA) at room temperature in dark. Negative controls were used for each donor sample to define TH+ and DAT+ expressing cells. For negative controls only secondary antibody staining was conducted and primary antibody staining with anti DAT and TH antibodies omitted. A washing step with 2 ml-s of flow cytometry buffer followed every staining procedure. Cells were re-suspended in flow cytometry buffer and analysed using LSRFortessa flow cytometer (BD Biosciences, CA, USA) and BD FACSDiva software (BD Biosciences, CA, USA). Experiments were compensated using single-colour compensation controls of SPHERO™ COMPtrol antibody capture beads (CMIgP-70-3K, Spherotech) with aforementioned fluorochrome conjugated antibodies, except anti rabbit and anti-mouse secondary antibodies were replaced with APC anti-human CD56 (392406, Biolegend) and BV241 anti CD127 (351310, Biolegend). 100 000 events were recorded for every sample. The gating strategy and optical detectors configuration are shown in a supplementary file (Table S1, Figure S1, Figure S2).

**Additional file 1: Table S1.** Configuration details of LSR Fortessa optical detectors.

| **Laser** | **Detector** | **LP Mirror** | **BP Filter** | **Intended Dye** |
| --- | --- | --- | --- | --- |
|  | A | 635 | 710/50 | - |
| 488-nm blue laser | B  C | 505 | 530/30  488/10 | FITC  Side scatter (SSC) |
|  | A | 670 | 710/40 | - |
|  | B | 630 | 670/30 | - |
| 405-nm violet laser | C | 600 | 610/20 | - |
|  | D | 535 | 540/30 | - |
|  | E | 505 | 525/50 | - |
|  | F | - | 440/40 | BV421 |
| 355-nm UV laser | A | 505 | 530/30 | - |
|  | B | - | 450/50 | BUV395 |
|  | A | 750 | 70/60 | - |
| 640-nm red laser | B | 690 | 730/45 | AF700 |
|  | C | - | 670/14 | APC |
|  | A | 750 | 780/60 | - |
|  | B | 685 | 710/50 | - |
| 561-nm YG laser | C | 635 | 670/30 | - |
|  | D | 600 | 610/20 | PE-CF594 |
|  | E | - | 586/15 | - |


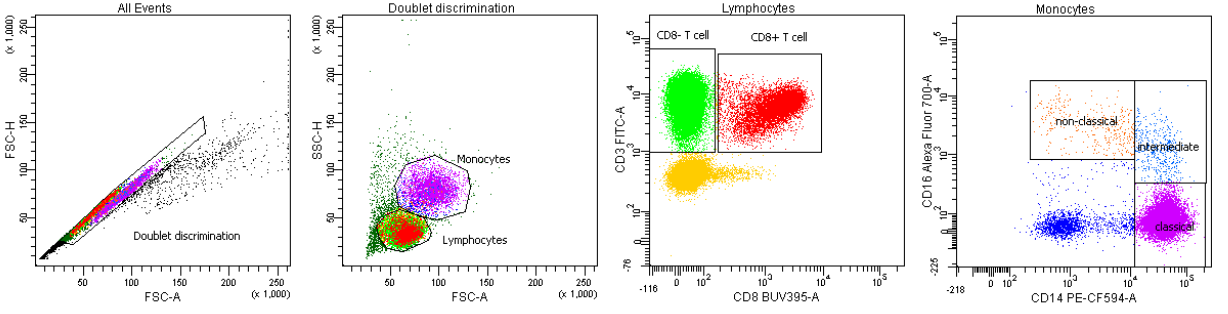


**Additional file 1: Figure S1.** Gating strategy for defining T-lymphocyte and monocyte subsets.

Firstly, singlets were selected to exclude doublets from further analysis based on forward scatter height (FSC-H) and forward scatter area (FSC-A). Next, lymphocyte and monocyte subsets were selected based on the side scatter height (SSC-H) and forward scatter area (FSC-A), describing their size and granularity, respectively. Finally, CD8^-^ and CD8^+^ T-cell subsets were defined based on their expression of CD8 and CD3 surface markers, non-classical, intermediate and classical monocyte subsets were detected based on their expression of CD16 and CD14.


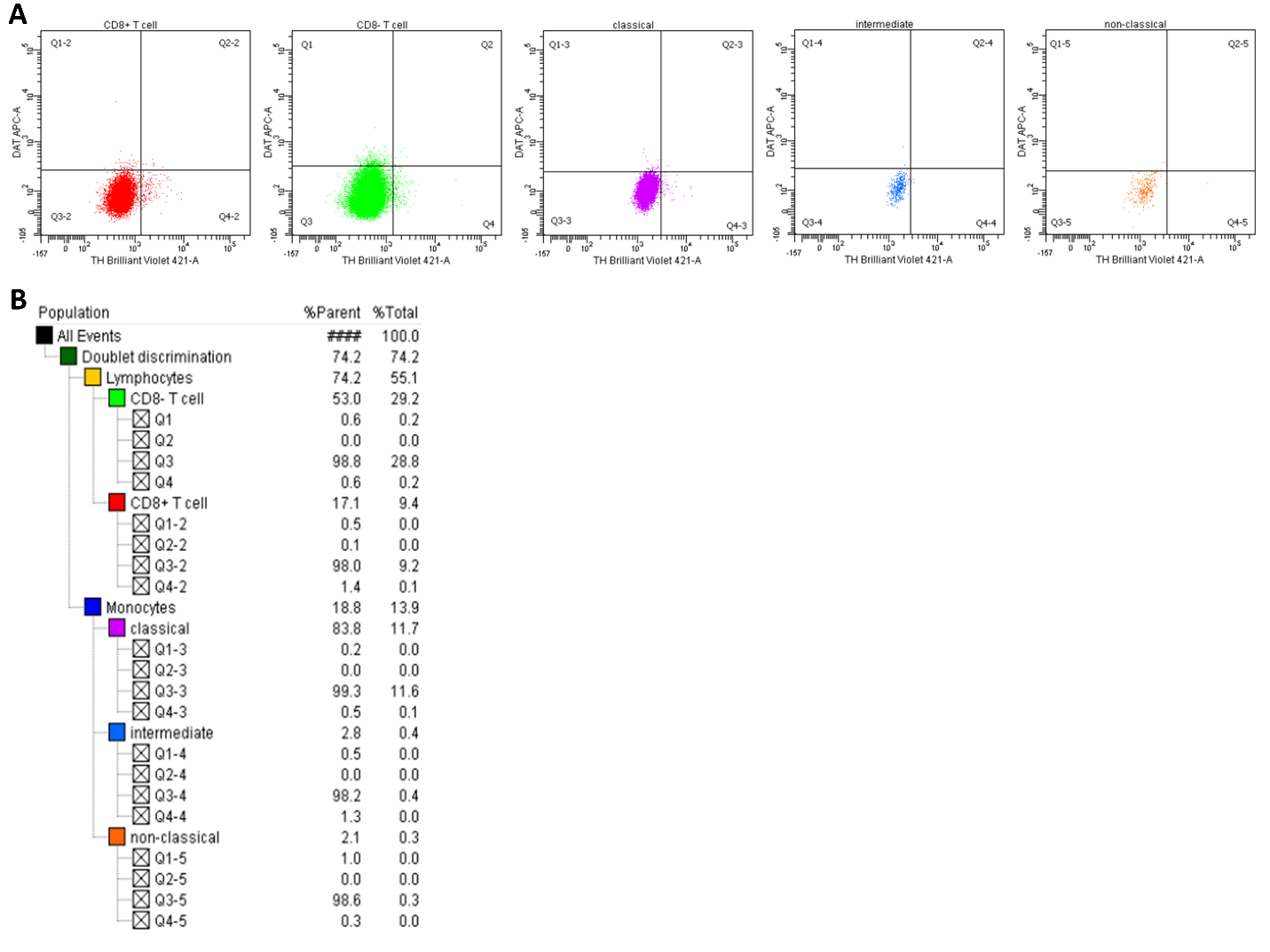


**Additional file 1: Figure S2.** Staining of tyrosine hydroxylase (TH) and dopamine transporter (DAT) in negative controls.

TH and DAT expressing cells were defined using a negative control stained with only secondary, but not primary antibodies for TH and DAT. A separate negative control was used for each donor sample. Only a marginal amount of cells show DAT and TH staining on negative controls (A, B).

***Western blot analysis***

Western blot analysis was conducted to evaluate the protein levels of TET1 and DNMT1 in PBMCs after repeated treatment with cocaine and amphetamine of PBMCs (n=8). RIPA buffer (Thermo Scientific, MA, USA) with protease inhibitor cocktail (Roche, Germany) was added to cells, which were lysed by pipetting and kept on ice for 20 min, before centrifugation for 20 min at 14 000 rpm. Supernatants were removed and protein levels were detected with RC DC Protein Assay Kit (Bio-Rad Laboratories, CA, USA). Cell lysates were diluted in 4x Laemmli sample buffer, containing dithiothreitol and heated at 70 °C for 10 min. 20 µg of protein was loaded on 7% minigels, gel electrophoresis was performed at 50 V until the samples ran out of stacking gel and 90 V onward on Bio-Rad system. Proteins were transferred from gel to 0.45 µm pore size Immobilon-FL PVDF membrane (Merck Millipore, MA, USA) in transfer buffer containing 15% methanol in 2.5 h. Membranes were blocked in Odyssey® blocking buffer (Li-Cor Biosciences, NE, USA) for 1 h and incubated with primary antibodies mouse anti-TET1 1:750 (GTX67420, Genetex, CA, USA) or rabbit anti-DNMT1 1:1000 (ab188453, Abcam, Cambridge, UK) for 72-96 hours in blocking buffer at +4C, following an incubation with secondary antibodies: Goat anti-Mouse IRDye® 800 CW 1:10000 (926-32210, Li-Cor Biosciences, NE, USA) and Goat anti-Rabbit IRDye® 800 (926-32211, Li-Cor Biosciences, NE, USA) for 1 hour at room temperature. For normalization of the protein levels, membranes were incubated with a rabbit anti β-actin 1:5 000 (926-42210, Li-Cor Biosciences, NE, USA) or mouse anti β-actin antibody 1:10 000 (926-42212, Sigma-Aldrich) for 12 hours at +4C, followed by incubation with Goat anti-Rabbit IRDye® 680 LT 1:10000 (926-68021, Li-Cor Biosciences, NE, USA) or Goat anti-Mouse IRDye® 680 LT 1:10000 (cat no 926-68020, Li-Cor Biosciences, NE, USA) in blocking buffer for 1 h at room temperature. Immunoreactive bands were detected by using Odyssey Infrared Imaging System (Odyssey CLx, Li-Cor Bioscienes, NE, USA). Optical density (OD) values were evaluated using ImageJ software. All OD values were normalized to the OD values of household protein β–actin.

***Illumina Next Generation Sequencing (NGS) Bioinformatics***

Raw data quality control was made with FastQC v.0.11.9 [1]. Data trimming was made with Trimmomatic-0.39 [2] in paired-end mode, removing max 3 low-quality base pairs from both the start and the end of the read. The quality control after trimming was made with FastQC v.0.11.9. Trimmed and filtered reads were aligned using Bismark-0.19.1 [3] to Human genome version 19 (hg19) in the paired-end mode [4]. Alignment quality and statistics were obtained with Picard-tools v.2.0.1 [5] package modules: CollectAlignmentSummaryMetrics, CollectGcBiasMetrics, CollectWgsMetrics, QualityScoreDistribution. The CpG site coverage was calculated with bedtools v2.27.0 (Quinlan and Hall, 2010). For methylation extraction and data quality statistics was used Bismark-0.19.1 Methylation Extractor module [3]. The R (3.6.0) package RnBeads 2.8.0 was used for downstream methylation analyses [6,7]. Gene Ontology (GO) Enrichment Analysis was conducted using a hypergeometric test that addresses the hierarchical structure of the ontology [8].

**Results**


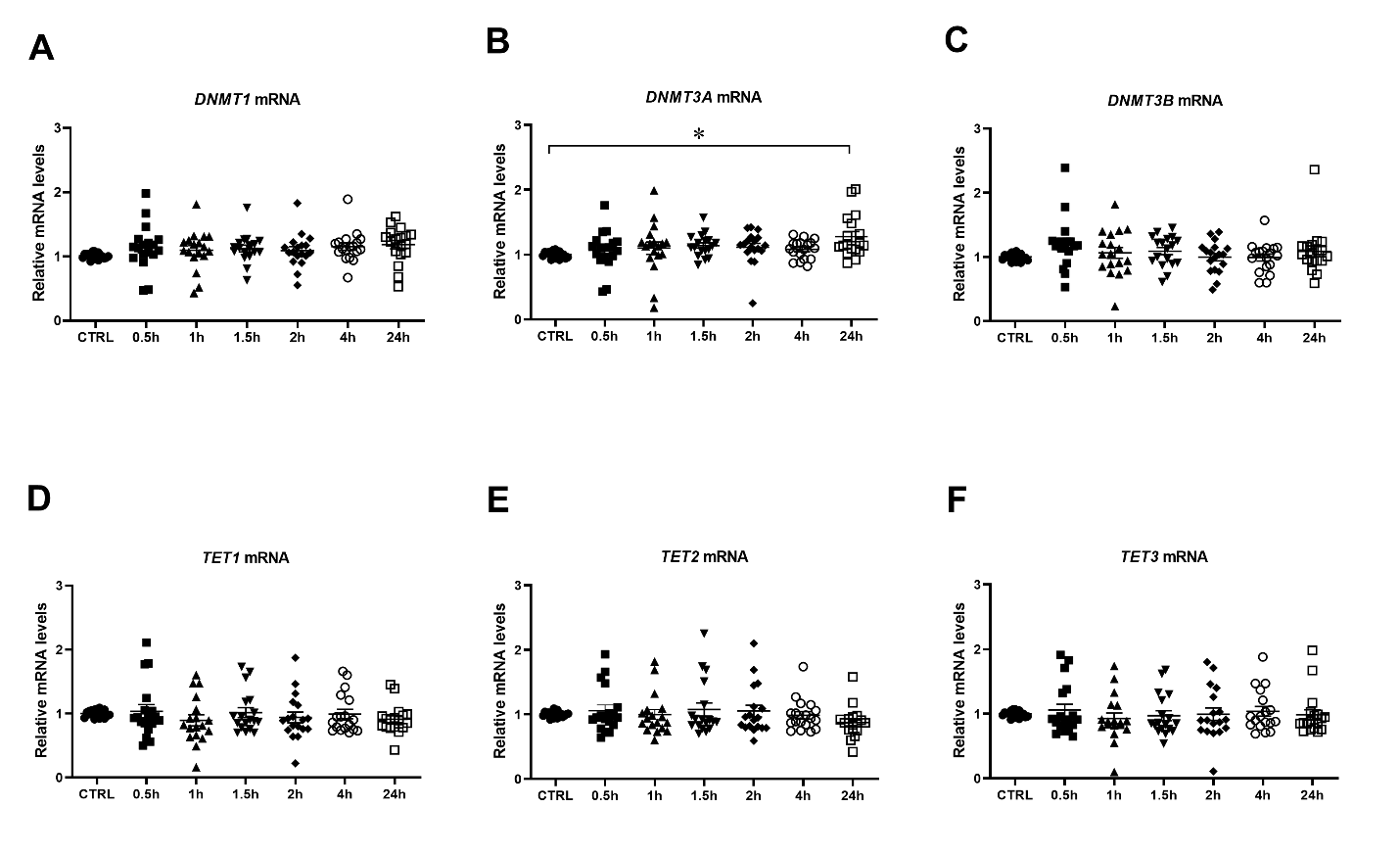


**Additional file 1: Figure S3.** Changes in the mRNA levels of *DNMT*s (A-C) and *TET1-3* (D-F), in response to acute amphetamine exposure *in vitro* in human peripheral blood mononuclear cells (PBMCs). One-way ANOVA, followed by Bonferroni post-hoc test; **p* < 0.05, *n* = 18 in all groups. CTRL= control. Error bars indicate SEM.


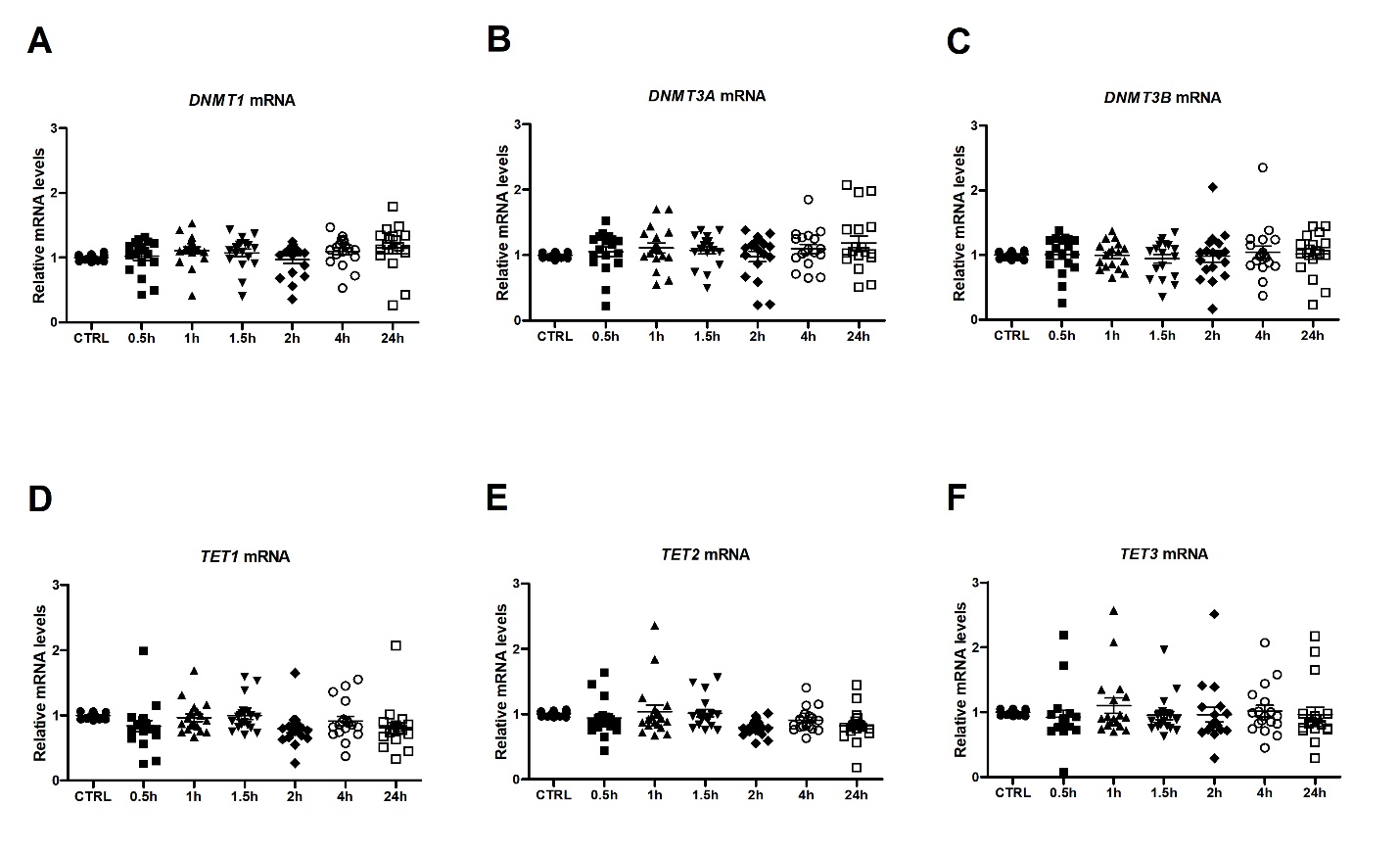


**Additional file 1: Figure S4.** Changes in the mRNA levels of *DNMT*s (A-C) and *TET1-3* (D-F) in response to acute cocaine exposure *in vitro* in human PBMCs. One-way ANOVA, followed by Bonferroni post-hoc test; *p* > 0.05, *n* = 18 in all groups. CTRL= control. Error bars indicate SEM.


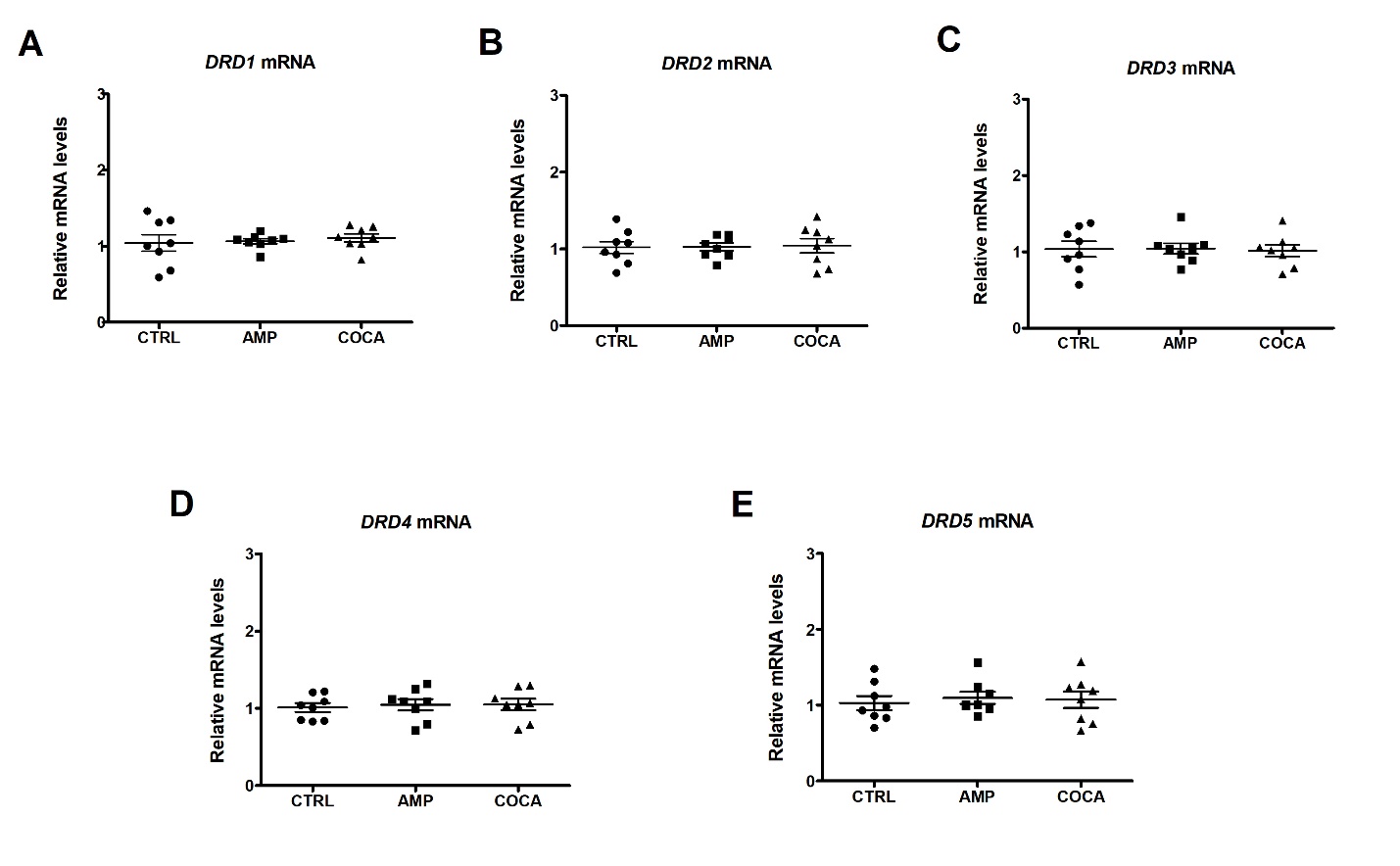


**Additional file 1: Figure S5.** Changes in the mRNA levels of *DRD1-DRD5* (A-E) in response to repeated amphetamine and cocaine exposure in human PBMCs. One-way ANOVA, followed by Bonferroni post-hoc test; *p* > 0.05, *n* = 8 in all groups. CTRL= control, AMP = amphetamine, COCA = cocaine. Error bars indicate SEM.


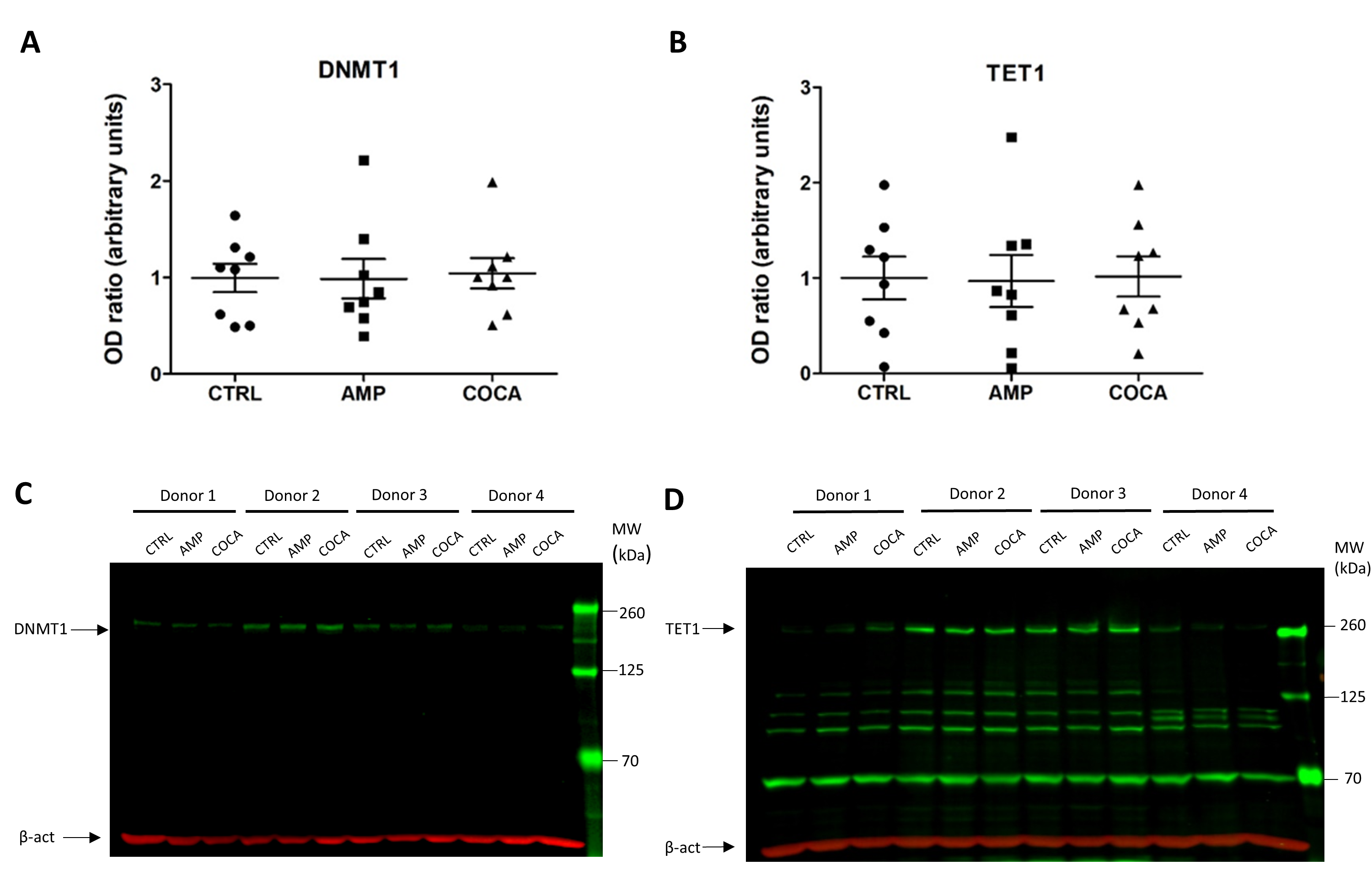


**Additional file 1: Figure S6.** Changes in DNMT1 (A) and TET1 (B) protein levels in response to repeated amphetamine and cocaine exposure in human PBMCs. One-way ANOVA, followed by Bonferroni post-hoc test; *p* > 0.05, *n* = 8 in all groups. Error bars indicate SEM. Full western blot images for quantification of protein levels of DNMT1 (C) and TET1 (D). Samples from four donors are represented on the images. CTRL= control, AMP = amphetamine, COCA = cocaine, OD = optical density, β-act – β-actin, MW=molecular weight.

**References**

1. Andrews S. A quality control tool for high throughput sequence data. [Internet]. 2010. Available from: https://www.bioinformatics.babraham.ac.uk/projects/fastqc/

2. Bolger AM, Lohse M, Usadel B. Trimmomatic: a flexible trimmer for Illumina sequence data. Bioinformatics. 2014;30:2114–20.

3. Krueger F, Andrews SR. Bismark: a flexible aligner and methylation caller for Bisulfite-Seq applications. Bioinformatics. 2011;27:1571–2.

4. Guo Y, Dai Y, Yu H, Zhao S, Samuels DC, Shyr Y. Improvements and impacts of GRCh38 human reference on high throughput sequencing data analysis. Genomics. 2017;109:83–90.

5. Broad Institute. Picard Toolkit [Internet]. 2019. Available from: http://broadinstitute.github.io/picard/

6. Assenov Y, Müller F, Lutsik P, Walter J, Lengauer T, Bock C. Comprehensive analysis of DNA methylation data with RnBeads. Nat Methods. 2014;11:1138–40.

7. Müller F, Scherer M, Assenov Y, Lutsik P, Walter J, Lengauer T, et al. RnBeads 2.0: comprehensive analysis of DNA methylation data. Genome Biology. 2019;20:55.

8. Falcon S, Gentleman R. Using GOstats to test gene lists for GO term association. Bioinformatics. 2007;23:257–8.
